# One size does not fit all: identifying clusters of physical activity, screen time, and sleep behaviour co-development from childhood to adolescence

**DOI:** 10.1186/s12966-020-00964-1

**Published:** 2020-05-11

**Authors:** François Gallant, Véronique Thibault, Jeffrey Hebert, Katie E. Gunnell, Mathieu Bélanger

**Affiliations:** 1 Université de Sherbrooke, Sherbrooke, Canada; 2 Centre de formation médicale du Nouveau-Brunswick, Moncton, Canada; 3 University of New Brunswick, Fredericton, Canada; 4 Murdoch University, Murdoch, Australia; 5 Carleton University, Ottawa, Canada; 6 Vitalité Health Network, Bathurst, Canada

## Abstract

**Purpose:**

Canada was the first to adopt comprehensive 24-h movement guidelines that include recommendations for physical activity, screen time and sleep to promote health benefits. No studies have investigated the concurrent development of these behaviours in youth. The objectives were to assess adherence to the Canadian 24-h movement guidelines for children and youth and estimate co-development of self-reported moderate-to-vigorous intensity physical activity (MVPA), screen time and sleep during 8-years from childhood to adolescence.

**Methods:**

Nine hundred and twenty three participants of the MATCH study self-reported their MVPA, screen time and sleep duration at least twice over 8 years. MVPA and screen time were measured three times per year (24 cycles), and sleep was measured once per year (8 cycles). Guideline adherence was dichotomised as meeting each specific health behaviour recommendation or not. Multi-group trajectory modeling was used to identify unique trajectories of behavioural co-development. Analyses were stratified by sex.

**Results:**

Between 10 and 39% of youth did not meet any recommendation at the various cycles of data collection. More than half of youth met only one or two recommendation, and roughly 5% of participants met all three recommendations at one or more study cycle throughout the 8 years of follow-up. Four different trajectories of behavioural co-development were identified for boys and for girls. For boys and girls, a complier (good adherence to the guideline recommendations; 12% boys and 9% girls), a decliner (decreasing adherence to the guideline recommendations; 23% boys and 18% girls) and a non-complier group (low adherence to the guideline recommendations; 42% boys and 42% girls) were identified. In boys, a MVPA-complier group (high MVPA-low screen time; 23%) was identified, whereas in girls a screen-complier group (moderate screen time-low MVPA; 30%) was identified.

**Conclusions:**

There is a need to recognise that variations from general trends of decreasing MVPA, increasing screen time and decreasing sleep exist. Specifically, we found that although it is uncommon for youth to adhere to the Canadian 24-h movement guidelines, some youth displayed a high likelihood of attaining one or multiple of the behavioural recommendations. Further, patterns of adherence to the guidelines can differ across different sub-groups of youth.

## Introduction

In 2016, Canada became the first country to adopt 24-h movement guidelines for children and youth that include recommendations for physical activity, screen time, and sleep to promote optimal development [1]. Specifically, these guidelines recommend that 5- to 17-year-olds accumulate at least 60 min of moderate-to-vigorous intensity physical activity (MVPA) and less than 2 h of recreational screen time per day and between 9 and 11 (5- to 13-year-olds) or 8 and 10 (14- to17-year-olds) hours of sleep per night [1]. These evidence-based recommendations resulted from a series of systematic reviews assessing the impact of each health-related behaviour on health outcomes [2–5].

Population-based studies show that youth typically experience declining physical activity [6–8], increasing screen time [9, 10], and decreasing sleep [11, 12] as they get older. However, recent studies have identified considerable between-individual variation in physical activity [13], sedentary time [14], and sleep [15] during childhood and adolescence. Given this between-individual variation, further studies identified subgroups of young people who follow developmental trajectories that differ from the average course. For example, although most young people experience a decrease in MVPA with age, some are consistently active [8, 16]. Studies investigating screen time behaviour report that most adolescents maintain or increase screen time, while others decrease screen time with age [17, 18]. With regard to sleep, one three-year study documented four declining trajectories of sleep time where most youth were classified as members of a low-normal (declining from 7.3 h to 6.8 h) or high-normal (8.2 h to 7.7 h) sleep duration trajectory [19]. These studies highlight that individuals follow different trajectories of specific health-related behaviours during childhood and adolescence; however, very few studies have investigated the co-development of these behaviours over time.

Of studies that have documented the co-development of health-related behaviours, most have only investigated two out of the three guideline behaviours. Kwon et al. (2015) found that most young people experience a decrease in MVPA and an increase in screen time with age, while a subgroup maintained about 50 min of daily MVPA and decreased their screen time [17, 20]. Another co-development study of physical activity and screen time found that children could be clustered in three different patterns: low physical activity/low screen time, increasing physical activity/low screen time, and low physical activity/increasing screen time [21]. To date, only one study has identified co-development trajectories of physical activity, sedentary behaviour, and sleep [22]. Using a sample of youth from two South-Africa cities, Hanson et al., (2019) demonstrated that while physical activity declined over time in both sexes, it was the only variable that was able to distinguish between the different groups of behaviour co-development. In addition, they only found one group in both girls and boys that maintained their physical activity levels over adolescence [22].

To date, no studies have attempted to investigate the co-development of physical activity, screen time, and sleep in youth as described in the Canadian 24-h movement guidelines. This knowledge is critical to identifying different subgroups of children and adolescents based on their developmental trajectories across the 24-h movement criteria. The identification of subgroups of children and adolescents will facilitate the development of tailored interventions aimed at improving compliance with health-behaviour guidelines. Therefore, the first objective of this study was to assess adherence with the Canadian 24-h Movement Guidelines for children and youth. The second objective was to identify common patterns in which MVPA, screen time, and sleep develop concurrently from childhood to adolescence.

## Methods

### Participants

We used data from cycles 1 to 24 of the Monitoring Activities of Teenagers to Comprehend Their Habits (MATCH) study, a prospective cohort study aimed at identifying determinants of health behaviours in children and adolescents. Detailed methodology is available elsewhere [23]. Briefly, 806 participants (ages 9 to 11) were recruited into the MATCH study from 17 schools in New Brunswick, Canada in 2011. Additional students from participating schools joined the study after the first year such that 938 children took part in at least one survey cycle of the study from September 2011 to June 2019. Participants were invited to participate in three survey cycles per school year. Only participants who participated in at least two survey cycles were included in the current analyses (*n* = 923). In a sensitivity analysis, the models were constructed with participants that participated in at least three survey cycles (*n* = 894) which led to no substantial differences compared to participants with at least two survey cycles (not presented). The MATCH study obtained ethical approval from the Université de Sherbrooke research ethics committee (#2012–321, 11–025). All participants provided written and informed assent and their parents provided written consent.

### Measures

#### MVPA

Participants reported MVPA in two items [24] at every survey cycle. After reading a preamble: “*Physical activity is any activity that increases your heart rate and makes you get out of breath some of the time. Physical activity can be done in sports, playing with friends, or walking to school. Some examples of physical activity are running, brisk walking, rollerblading, biking, dancing, skateboarding, swimming, soccer, basketball, hockey, and skiing.*” they indicated the number of days they engaged in MVPA for at least 60 min in (1) a typical week and (2) the past week. Response options ranged from 0 to 7 (days). As recommended when using this scale [24], the average of the two items was used to create an overall MVPA score for analyses. Specifically, participants were classified as meeting the physical activity recommendation if they reported MVPA on at least 7 (rounded values of ≥6.5) days per week at that cycle. Using this approach in previous MATCH analyses yielded estimates that approach those obtained through objective measures among a representative sample of same-aged Canadian youth [25]. This measure has acceptable test-retest reliability (ICC_1,1_ = 0.77) and is associated with accelerometer-measured MVPA (r = 0.40) among 12-year olds [24].

#### Screen time

Participants reported screen time using three items [26] at every survey cycle. Explicitly, they reported (1) on an average weekday and (2) on an average weekend day, how many hours they spent (a) watching TV & videos, (b) using a computer, iPad, tablet (not for homework), and (c) playing video games, such as XBOX, Wii, and PlayStation as well as on iPod, iPad, tablet or cell phone. Response options were: *0 h, ½ hour, 1 h, 2 h, 3 h, 4 h or 5h hours*. For participants reporting *5h hours* of screen time, responses were set at 5 h. Average daily screen time was computed using the following formula: average daily screen time = [(5* sum of indicators on weekdays) + (2* sum of indicators on weekends)]/7. Participants were then classified as meeting the screen time recommendations if they reported 2 h or less of daily screen time on average. This measure has demonstrated acceptable test-retest reliability (r = 0.60 to r = 0.80) [26]. Another similar self-reported questionnaire on screen time also showed good test-retest reliability (κ > 0.70) on weekdays and weekends measures for television watching, computer games, console games and internet use [27]. Results from past research using similar screen time measures suggest that the measures have good convergent validity or discriminant validity [28]. Moreover, we previously described having to modify this measure during the study to reflect changes in screen-based technologies (i.e., examples of screen devices were added) and reported that it is still appropriate to conduct longitudinal assessments of screen time following the adjustments [29].

#### Sleep

Participants reported sleep duration using four items regarding their usual bed and wake times on weekdays and weekend days [30] at cycles 1, 2, 4, 7, 10, 13, 16, 19 and 22 (i.e. once per year after cycle 2). All response options were provided in half hour increments. For weekday bed times, response options ranged from *7:00 pm or earlier* to *12:30 am or later.* For weekday wake time, response options ranged from *5:00 am or earlier* to *8:30 am or later*. Response options for weekend bed times were the same as weekday bed times, but more wake time options were given, so that the last option was *10:30 am or later* (instead of *8:30 am or later*) on weekends. Sleep duration on usual weekdays and weekend days were obtained by subtracting wake time from bed time. Mean nightly sleep duration was calculated as = [(5* weekday sleep duration) + (2* weekend day sleep duration)]/7. Participants 13 years or younger were classified as meeting the sleep recommendation if average sleep duration was between 9 and 11 h. Similarly, when participants were older than 13 years old, they were classified as meeting the sleep guidelines if average sleep duration was between 8 and 10 h. The sleep duration scale has acceptable internal consistency (α = 0.75) and is associated with diary (r = 0.61 week-night; r = 0.38 weekend-night) and accelerometer (r = 0.53 week-night; r = 0.31 weekend-night) measures [30, 31].

### Data analysis

To answer objective one, descriptive statistics were used to assess adherence to the Canadian 24-h movement guidelines in three ways. First, we used t-tests comparing both sexes at each survey cycle to describe differences between mean number of days per week participants attained ≥1 h/day MVPA, daily screen time use, and nightly sleep duration. Second, we used χ^2^ tests to compare the proportion of children who met guideline recommendations for each behaviour independently between sexes. Third, we examined the number of participants meeting none, one, two, or all three recommendations when all three behaviours were measured concurrently. All analyses were conducted in SAS (version 9.4) and two-sided alpha level was set to 5 %. To answer objective two, which was to identify patterns of co-development of MVPA, screen time, and sleep, we used the PROC TRAJ procedure extension for SAS to construct group-based multi-trajectory models, which allow for the identification of latent classes (i.e. groups of individuals following similar development of behaviours over time) through a special application of finite mixture modeling. First, we constructed models for each behaviour (i.e., physical activity, screen time, and sleep) separately to understand occurring patterns over time. Next, this information was used to inform model selection for the multi-trajectory models wherein all behaviours were modeled simultaneously. The multi-trajectory model was constructed as a function of age to describe the probability of adhering to each health behaviour concurrently over time [32]. Model selection was conducted in two steps. The first step of model selection was to identify the number of trajectory groups within the data and was based on the Bayesian information criterion (BIC) and substantive significance [33]. Specifically, we identified the number of latent classes using the BIC. Then we determined if different latent classes pragmatically expressed different trajectories (i.e. an increase in the number of classes did not provide a substantially different group of individuals). In all analyses we aimed to obtain minimal class sizes of at least 5% of the sample. Second, we tested the polynomial order of each latent class to determine the pattern of change over time (linear, quadratic, or cubic). Once the final models were selected, their adequacy was assessed by verifying that the average posterior probability of group membership was ≥70% and that the odds of correct classification was ≥5 for each group. Further, we assessed the precision of the estimated probability and the similarity between the estimated probability of the trajectory group and the proportion assigned to the group, as suggested by Nagin (2005) [33]. Following the identification of the trajectory groups, each group was assigned a label characterising their pattern of adherence to the recommendations included in the 24-h movement guideline. Because of known differences for health-related behaviours between sexes [6, 34], the multi-trajectory models were stratified by sex.

## Results

### Descriptive analysis of behaviours

Participants (*n* = 923; 55% girls) initially aged 10.3 (SD, 0.6) years, provided data at least twice over 24 data collection cycles spanning 8 years and were included in the analysis. The median number of survey cycles participants took part in was 13 (IQR, 8–19). Generally, MVPA and screen time followed quadratic patterns over the study duration (i.e. MVPA and screen time followed an inverted U-shape pattern, where participants reported an increase in both behaviours until a high point (MVPA: cycle 9; screen time: cycle 14) and then declined until cycle 24). For girls and boys, average MVPA levels increased slightly for the first 9 survey cycles (girls: 4.2 to 4.7 days per week; boys: 4.7 to 5 days per week) declining for the remainder of the study (Table 1). By cycle 16, MVPA levels were lower than those reported at baseline (girls: 4.1 days per week; boys: 4.4 days per week). In contrast, girls and boys reported an increase in screen time over the first 14 survey cycles (girls: 2.7 to 5.8 h per day; boys: 2.9 to 6.3 h per day), after which screen time declined. Despite this decline, screen time levels remained higher at cycle 24 than at baseline (girls: 4 h per day; boys: 4.9 h per day). On average, sleep time decreased in a linear fashion for both sexes over the 8 year study duration (girls: 9.7 to 8.5 h per night; boys: 9.4 to 8.2 h per night). Boys generally reported more MVPA and screen time than girls for most cycles. Sleep duration was similar in girls and boys, except for the first year of the study, where girls reported greater sleep time.

**Table 1:** Average amount of physical activity, screen time and sleep for participants in the MATCH study over 24 data collection cycles

	Girls						Boys					
Cycle	n	MVPA (days/week: attaining ≥1 h/day) (mean, 95% CI)	n	Screen time (hours/day) (mean, 95% CI)	n	Sleep (hours/day) (mean, 95% CI)	n	MVPA (days/week attaining ≥1 h/day) (mean, 95% CI)	n	Screen time (hours/day) (mean, 95% CI)	n	Sleep (hours/day) (mean, 95% CI)
1	328	**4.23 (4.03–4.43)**	303	2.70 (2.48–2.2.92)	201	**9.65 (9.48–9.82)**	265	4.68 (4.46–4.90)	242	2.99 (2.70–3.28)	159	9.37 (9.14–9.60)
2	406	4.28 (4.10–4.46)	397	**2.91 (2.71–3.11)**	346	**9.81 (9.71–9.91)**	314	4.49 (4.28–4.70)	311	3.25 (3.00–3.50)	259	9.64 (9.50–9.78)
3	402	**4.52 (4.35–4.69)**	396	**3.28 (3.03–3.52)**			331	4.86 (4.68–5.06)	327	4.40 (3.99–4.81)		
4	392	4.54 (4.37–4.71)	392	**3.33 (3.09–3.57)**	392	**9.69 (9.61–9.77)**	294	4.63 (4.43–4.85)	294	4.72 (4.35–5.09)	286	9.48 (9.36–9.60)
5	386	4.43 (4.25–4.60)	386	**3.67 (3.39–3.95)**			290	4.55 (4.34–4.76)	288	5.17 (4.78–5.55)		
6	344	**4.55 (4.36–4.74)**	346	**3.58 (3.29–3.88)**			275	4.89 (4.67–5.11)	275	5.14 (4.69–5.59)		
7	360	4.81 (4.64–4.98)	364	**3.28 (3.02–3.54)**	365	9.34 (9.24–9.44)	285	5.00 (4.79–5.21)	287	4.87 (4.48–5.26)	283	9.20 (9.08–9.33)
8	353	4.52 (4.33–4.70)	358	**3.53 (3.26–3.81)**			276	4.58 (4.34–4.82)	275	5.34 (4.91–5.78)		
9	343	**4.67 (4.48–4.86)**	342	**4.77 (4.42–5.12)**			263	4.98 (4.76–5.21)	263	5.65 (5.21–6.10)		
10	320	**4.56 (4.36–4.75)**	318	**5.11 (4.73–5.48)**	315	9.08 (8.97–9.18)	245	4.92 (4.69–5.16)	244	5.87 (5.39–6.36)	238	9.01 (8.90–9.12)
11	305	4.53 (4.32–4.74)	305	**5.24 (4.86–5.61)**			225	4.73 (4.46–5.00)	225	6.04 (5.54–6.53)		
12	303	4.60 (4.40–4.80)	307	**5.22 (4.83–5.60)**			232	4.82 (4.55–5.09)	233	5.89 (5.40–6.39)		
13	304	**4.39 (4.17–4.60)**	302	**5.24 (4.86–5.62)**	301	8.73 (8.62–8.84)	228	4.75 (4.47–5.04)	227	6.13 (5.63–6.64)	216	8.69 (8.52–8.85)
14	266	4.64 (4.43–4.85)	273	5.76 (5.35–6.17)			199	4.75 (4.47–5.03)	206	6.30 (5.83–6.77)		
15	250	**4.27 (4.03–4.50)**	252	5.08 (4.69–5.47)			197	4.76 (4.47–5.05)	191	5.72 (5.20–6.25)		
16	239	4.13 (3.87–4.38)	237	**4.64 (4.23–5.04)**	236	8.54 (8.39–8.70)	174	4.37 (4.05–4.70)	173	5.48 (4.94–6.01)	175	8.51 (8.30–8.71)
17	218	4.08 (3.81–4.35)	241	4.24 (3.85–4.62)			172	4.37 (4.04–4.70)	193	4.78 (4.31–5.26)		
18	214	**3.61 (3.33–3.89)**	212	4.45 (4.01–4.89)			156	4.14 (3.80–4.49)	156	5.03 (4.49–5.56)		
19	205	**3.64 (3.35–3.93)**	203	**4.46 (4.01–4.91)**	204	8.37 (8.22–8.51)	145	4.24 (3.86–4.63)	145	5.43 (4.84–6.01)	148	8.24 (8.05–8.43)
20	207	**3.86 (3.58–4.14)**	207	**4.61 (4.19–5.03)**			152	4.35 (4.01–4.69)	155	5.69 (5.16–6.23)		
21	201	**3.70 (3.43–3.98)**	199	**4.58 (4.14–5.01)**			142	4.20 (3.83–4.57)	143	5.21 (4.63–5.79)		
22	117	4.00 (3.60–4.40)	115	4.23 (3.66–4.81)	118	8.45 (8.29–8.61)	85	4.29 (3.82–4.77)	86	5.10 (4.42–5.57)	81	8.18 (7.95–8.41)
23	107	3.87 (3.46–4.27)	107	4.21 (3.62–4.80)			75	3.86 (3.33–4.39)	75	4.96 (4.21–5.71)		
24	97	3.89 (3.51–4.26)	97	4.01 (3.44–4.57)			68	3.69 (3.12–4.26)	67	4.88 (4.20–5.56)		

Results are presented as means and 95% confidence intervals; Sleep was measured in cycles 1, 2, 3, 7, 10, 13, 16, 19, 22

*MVPA* Moderate-to-vigorous physical activity

Bold represents different than boys (t test, *p* < 0.05)

### Adherence to the 24-h movement guidelines

The proportion of participants meeting the MVPA recommendation increased over the first 9 survey cycles (girls: 16.2 to 20.7%; boys: 23.4 to 32.7%) before decreasing for both sexes (at cycle 24, 9.3% of girls and 14.7% of boys met the MVPA guidelines). At most cycles, a greater proportion of boys met the MVPA recommendation than girls (Table 2). Overall, a decrease in the proportion of participants who met the screen time recommendation was noted, with nearly half of participants meeting the recommendation at baseline (47.9% of girls; 44.6% of boys) compared to 18% for boys and 27% for girls in cycle 24. For half of the cycles, a larger proportion of girls met the screen time recommendation compared to boys. Sleep recommendation had the highest probability of being met with up to 80% of participants reporting adherence during at least one survey cycle. However, the proportion of participants adherent to the sleep recommendation decreased over time (cycle 1: girls 81.1%; boys 73.6%) and reached a low of 43% for girls and boys in cycle 13 (i.e. their last year of the children-specific recommendation). No differences were found between the proportion of girls and boys meeting the sleep recommendation.

**Table 2:** Proportions of participants in the MATCH study meeting the specific recommendations of the 24-h Movement Guidelines over 24 data collection cycles

	Girls						Boys					
Cycle	n	MVPA (%, 95 CI)	n	Screen time (%, 95 CI)	n	Sleep (%, 95 CI)	n	MVPA (%, 95 CI)	N	Screen time (%, 95 CI)	n	Sleep (%, 95 CI)
1	328	**16.2 (12.6–20.5)**	303	47.9 (42.3–53.5)	201	81.1 (75.1–85.9)	265	23.4 (18.7–28.9)	242	44.6 (38.5–50.9)	159	73.6 (66.2–79.8)
2	406	**16.3 (13.0–20.2)**	397	42.3 (37.6–47.2)	346	81.5 (77.1–85.2)	314	22.3 (18.0–27.2)	311	37.6 (32.4–43.1)	259	76.5 (70.9–81.2)
3	402	**17.7 (14.3–21.7)**	396	**40.2 (35.4–45.1)**			331	26.0 (21.6–31.0)	327	26.0 (21.5–31.0)		
4	392	**17.6 (14.2–21.7)**	392	**36.2 (31.6–41.1)**	392	80.4 (76.1–84.0)	295	25.9 (21.2–31.1)	294	22.5 (18.1–27.6)	286	75.2 (69.9–79.8)
5	386	17.4 (13.9–21. 5)	386	**31.1 (26.7–35.9)**			290	21.4 (17.1–26.5)	288	16.7 (12.8–21.4)		
6	344	**18.9 (15.1–23.4)**	346	**37.6 (32.6–42.8)**			275	31.3 (26.1–37.0)	275	22.6 (18.0–27.8)		
7	360	**21.1 (17.2–25.6)**	364	**40.1 (35.2–45.2)**	365	71.5 (66.7–75.9)	285	31.2 (26.1–36.8)	287	21.6 (17.2–26.7)	283	69.6 (64.0–74.7)
8	353	**17.6 (14.0–21.9)**	358	**36.6 (31.8–41.7)**			276	25.7 (20.9–31.2)	275	15.6 (11.8–20.4)		
9	343	**20.7 (16.8–25.3)**	342	22.5 (18.4–27.2)			263	32.7 (27.3–38.6)	263	17.1 (13.0–22.1)		
10	320	**17.5 (13.7–22.0)**	318	17.6 (13.8–22.2)	315	66.7 (61.3–71.6)	245	29.8 (24.4–35.8)	244	12.3 (8.8–17.0)	238	64.3 (58.0–70.1)
11	305	**19.3 (15.3–24.2)**	305	**18.0 (14.1–22.7)**			225	28.9 (23.4–35.1)	225	10.7 (7.3–15.4)		
12	303	**18.5 (14.5–23.2)**	307	15.3 (11.7–19.8)			232	32.8 (27.0–39.0)	233	13.3 (9.5–18.3)		
13	304	**17.4 (13.6–22.1)**	302	**19.1 (15.2–24.0)**	301	42.9 (37.4–48.5)	228	34.7 (28.8–41.0)	227	11.0 (7.6–15.8)	216	42.6 (36.2–49.3)
14	266	**18.1 (13.9–23.1)**	273	12. 5 (9.1–16.9)			199	29.7 (28.8–41.0)	206	8.3 (5.2–12.8)		
15	250	**13.6 (9.9–18.4)**	252	17.9 (13.6–23.1)			197	29.4 (23.5–36.2)	191	14.7 (10.3–20.4)		
16	239	**15.1 (11.1–20.2)**	237	21.9 (17.1–27.6)	236	76.3 (70.5–81.3)	174	24.7 (18.9–31.6)	173	16.2 (11.4–22.4)	175	70.9 (63.7–77.1)
17	218	**15.6 (11.4–21.0)**	241	27.0 (21.7–32.9)			172	27.3 (21.2–34.4)	193	25.9 (20.2–32.5)		
18	214	**10.8 (7.3–15.6)**	212	26.9 (21.4–33.3)			156	20.5 (14.9–27.5)	156	19.2 (13.8–26.1)		
19	205	**10.7 (7.2–15.7)**	203	**26.1 (20.6–32.6)**	204	64.7 (57.9–70.9)	145	24.1 (17.9–31.7)	145	15.9 (10.8–22.7)	148	65.5 (57.6–72.7)
20	207	**13.5 (9.5–18.9)**	207	**23.2 (18.0–29.4)**			152	23.0 (17.1–30.3)	155	11.0 (7.0–16.9)		
21	201	12.4 (8.6–17.7)	199	21.6 (16.5–27.8)			142	19.7 (14.0–27.0)	143	18.9 (13.3–26.1)		
22	117	18.0 (12.1–25.9)	115	27.8 (20.5–36.6)	118	70.3 (61. 6–77.8)	85	23.5 (15.8–33.6)	86	19.8 (12.7–29.4)	81	63.0 (52.1–72. 7)
23	107	15.0 (9.4–22.9)	107	**29.9 (22.1–39.2)**			75	17.3 (10.4–27.4)	75	13.3 (7.4–22.8)		
24	97	9.3 (5.0–16.7)	97	26.8 (19.0–36.4)			68	14.7 (8.2–25.0)	67	17.9 (10.6–28.8)		

Results are presented as proportions and 95% confidence intervals; Sleep was measured in cycles 1, 2, 3, 7, 10, 13, 16, 19, 22

*MVPA* Moderate-to-vigorous physical activity;

Bold represents different than boys (χ^2^ p < 0.05)

The adherence to all 24-h movement guidelines was evaluated at 9 survey cycles (i.e., when all three behaviours were measured concurrently). At these cycles, the proportion of participants who did not meet any of the three recommendations ranged from 10 to 39% in girls and 12 to 36% in boys (Table 3). Approximately half of participants (44 to 59% for girls and 40 to 59% for boys) met one recommendation, around one quarter (14 to 38% for girls and 18 to 40% for boys) met two recommendations and less than 10% (3 to 9% for girls and 2 to 10% for boys) met all three recommendations at the various survey cycles.

**Table 3:** Recommendation adherence for cycles when MVPA, screen time and sleep were measured

		Number of recommendations met (%, 95 CI)			
Cycle	Sex	0	1	2	3
1	Girls (*n* = 199)	9.55 (6.49–12.60)	46.73 (41.55–51.92)	38.19 (33.14–43.24)	5.53 (3.15–7.90)
	Boys (*n* = 157)	11.46 (8.16–14.77)	39.49 (34.41–44.57)	39.49 (34.41–44.57)	9.55 (6.50–12.61)
2	Girls (*n* = 339)	10.62 (8.12–13.12)	46.40 (42.86–50.95)	34.81 (30.94–38.67)	7.67 (5.51–9.83)
	Boys (*n* = 245)	12.24 (9.59–14.90)	50.20 (46.15–54.26)	30.20 (26.48–33.93)	7.35 (5.23–9.46)
4	Girls (*n* = 388)	12.89 (10.35–15.42)	47.16 (43.39–50.94)	32.22 (28.68–35.75)	7.73 (5.71–9.75)
	Boys (*n* = 284)	15.49 (12.76–18.23)	51.41 (47.63–55.19)	27.11 (23.75–30.47)	5.99 (4.19–7.78)
7	Girls (*n* = 359)	13.65 (10.99–16.31)	48.75 (44.87–52.62)	28.69 (25.18–32.20)	8.91 (6.70–11.12)
	Boys (*n* = 280)	17.50 (14.55–20.45)	50.00 (46.12–53.88)	27.14 (23.69–30.59)	5.36 (3.61–7.10)
10	Girls (*n* = 309)	23.30 (19.75–26.85)	54.69 (50.51–58.87)	19.09 (15.79–22.39)	2.91 (1.50–4.32)
	Boys (*n* = 236)	24.58 (20.96–28.19)	48.73 (44.53–52.92)	22.03 (18.55–25.51)	4.66 (2.89–7.32)
13	Girls (*n* = 299)	39.46 (35.24–43.69)	43.81 (39.53–48.10)	14.05 (11.05–17.05)	2.68 (1.28–4.07)
	Boys (*n* = 216)	35.65 (31.51–39.78)	44.44 (40.15–48.74)	17.59 (14.30–20.88)	2.31 (1.02–3.61)
16	Girls (*n* = 233)	16.31 (12.68–19.94)	58.80 (53.96–63.63)	19.74 (15.83–23.65)	5.15 (2.98–7.32)
	Boys (*n* = 165)	16.97 (13.28–20.66)	58.79 (53.95–63.62)	18.79 (14.95–22.63)	5.45 (3.22–7.69)
19	Girls (*n* = 199)	24.62 (20.02–29.22)	50.75 (45.42–56.09)	21.61 (17.21–26.00)	3.02 (1.19–4.84)
	Boys (*n* = 138)	21.01 (16.66–25.36)	55.80 (50.49–61.10)	19.57 (15.33–23.80)	3.62 (1.63–5.62)
22	Girls (*n* = 114)	20.18 (14.54–25.81)	47.37 (40.36–54.38)	28.95 (22.58–35.31)	3.51 (0.93–6.09)
	Boys (*n* = 81)	27.16 (20.92–33.40)	43.21 (36.26–50.16)	25.93 (19.78–32.08)	3.70 (1.05–6.35)

Results are presented as proportions and 95% confidence intervals

### Multi-trajectory analysis

In both girls and boys (Appendix), a four-group model characterizing movement behaviour co-development emerged as the best fitting model since BIC improved only marginally and the additional groups emerging in the five- and six-group models were not pragmatically different from the other groups. Groups were characterized by their differences in the probability of adherence to the MVPA and screen time recommendations, while sleep behaviour provided little discriminatory information to distinguish between subgroups.

#### Girls

**Fig. 1:**
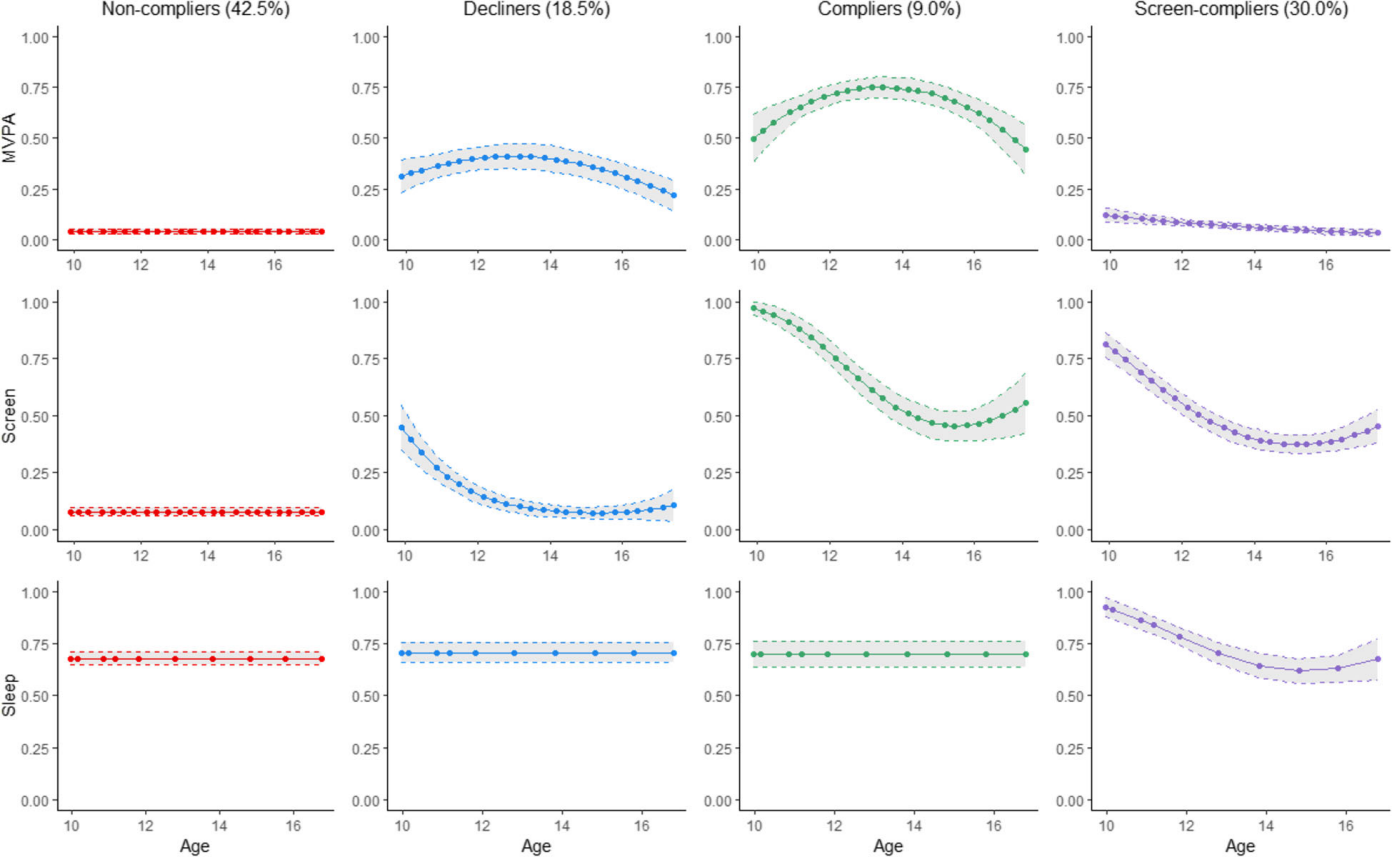
Multi-trajectory modeling of probability of attaining each 24-h movement guideline recommendation among girls in the MATCH study. MATCH: Monitoring Activities of Teenagers to Comprehend their Habits; MVPA: Moderate-to-vigorous physical activity; Solid lines represent the probability of meeting each movement guideline recommendation over time. Dashed line represents the 95% confidence interval

The largest subgroup (42.5%) was labeled *non-compliers* because they were characterized by a consistently low probability of attaining the MVPA and the screen time recommendations over time (Fig. 1). One subgroup (18.5%) was labeled *decliners* owing to their declining probability over time of meeting both MVPA and screen time recommendations. A smaller subgroup (9.0%) was labeled *compliers* because of their moderate probability of attaining both MVPA and screen time recommendations over time. Finally, another subgroup (30.0%) were labeled *screen-compliers* because they were characterized by a low probability of attaining the MVPA recommendation and moderate probability of attaining the screen time recommendation.

#### Boys

**Fig. 2:**
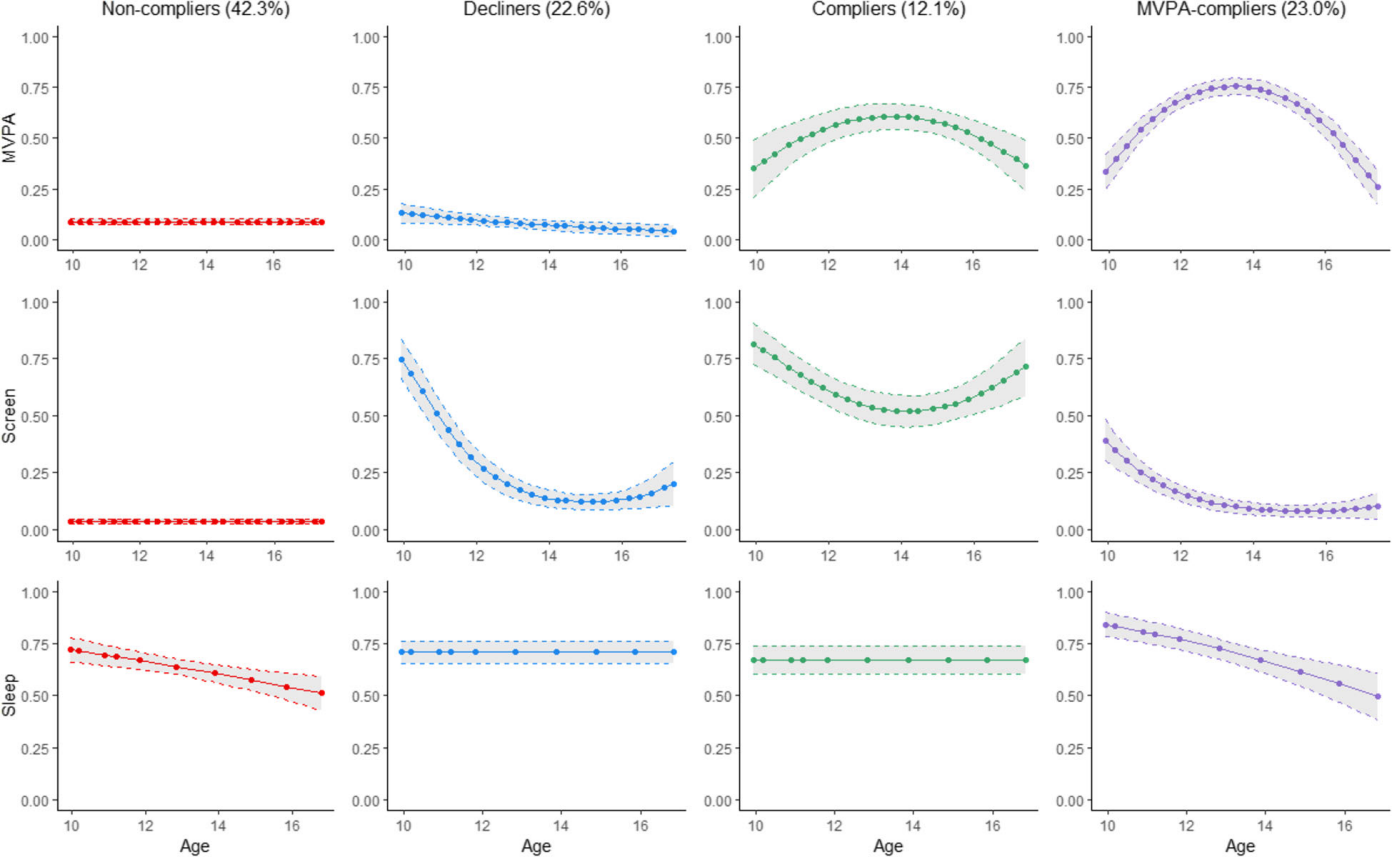
Multi-trajectory modeling of probability of attaining each 24-h movement guideline recommendation among boys in the MATCH study. MATCH: Monitoring Activities of Teenagers to Comprehend their Habits; MVPA: Moderate-to-vigorous physical activity; Solid lines represent the probability of meeting each movement guideline recommendation over time. Dashed line represents the 95% confidence interval

Of the four groups that emerged for boys, three were similar to the first three groups described above for girls. Specifically, the largest group among boys (42.3%) was labeled *non-compliers*, because they were characterized by a consistently low probability of attaining the MVPA and screen time recommendations (Fig. 2). One subgroup (22.6%) was labeled *decliners*, because of their consistently low and declining probability of attaining both the MVPA and screen time recommendations. A smaller group (12.1%) was labeled *compliers* and was represented by a moderate probability of attaining the MVPA recommendation and a high probability of attaining screen time recommendation. The last subgroup (23%) was labeled *MVPA-compliers*, because they were characterized by a high probability of attaining the MVPA recommendation and a low and declining probability of attaining the screen time recommendation.

## Discussion

In this study we described the evolution of adherence to the 24-h movement guidelines over 8 years in a sample of girls and boys. Whereas a large proportion of youth met one recommendation, roughly 5 % met all three. Our results also indicate that boys and girls follow four different trajectories of behaviour co-development over time. Only MVPA and screen time behaviours allowed to differentiate between trajectories. In both sexes, a *complier* group (moderate probability of attaining the MVPA recommendation and a high probability of attaining screen time recommendation), a *non-complier* group (consistently low probability of attaining the MVPA and screen time recommendations), and a *decliner* group (consistently low and declining probability of attaining both the MVPA and screen time recommendations) were identified. The fourth trajectory group differed between sexes. In girls, we identified a *screen-complier* group (low probability of attaining the MVPA recommendation and moderate probability of attaining the screen time recommendation) and a *MVPA-complier* group (high probability of attaining the MVPA recommendation and a low and declining probability of attaining the screen time recommendation) in boys.

Canadian youth may be missing out on the beneficial dose-response relationship between health behaviours and health outcomes [35]. Our results are consistent with two nationally representative cross-sectional studies of Canadian youth. In one cross-sectional study of approximately 20,000 Canadians aged 10-to 17-year-old, 1 in 5 (20.9%) of participants failed to adhere to any guideline recommendation, (51.1%) adhered to one recommendation, one quarter (25.3%) of participants adhere to two recommendations, and 2.6% met all three recommendations [36]. Similarly, the other cross-sectional analysis of 3111 Canadian youth found that 17.1% of participants aged 12 to 17 years met no recommendation, half (50.6%) of participants met one recommendation, 26.8% met two recommendations and 5.5% met all three recommendations [37]. Although our study is the first longitudinal analysis of the 24-h movement guidelines for youth in Canada, the consistency of our results with previous studies indicate that it is very uncommon for Canadian youths to adhere to the Canadian 24 h-movement guidelines. These results highlight the importance of future behaviour-change research aimed at improving these critical health behaviours in young people.

This is the first study to describe the co-development of MVPA, screen time, and sleep from childhood to late adolescence in North America. In a study from South-Africa [22], Hanson et al., (2019) found that physical activity trajectories, but not sedentary behaviour or sleep distinguished developmental groups over adolescence. This is different from our study, where we found that MVPA and screen time contributed to differentiating developmental groups of girls and boys. Differences in the operationalization of physical activity and sedentary behaviour variables might have led to the discrepancies in findings [22]. For example, their measure of physical activity comprised of time spent in various physical activity domains, including walking, informal physical activity, and organized sports, while we only measured attainment of the MVPA recommendation. Similarly, whereas Hanson et al., (2019) measured sedentary behaviour, which included screen time, but also reading, drawing, homework, and playing an instrument, we only investigated attainment of the screen time recommendations as defined in the Canadian 24-h movement guidelines. In addition, differences in findings between the two studies may be related to geographical and socioeconomic differences between the study populations.

In the multi-trajectory analysis, three of the four groups were similar across sexes. The most prevalent subgroup among girls and boys was described as *non-compliers* with the MVPA and screen time recommendations (i.e. low MVPA and high screen time). In contrast, the smallest subgroup for both sexes was characterized as *compliers* with both the MVPA and screen time recommendations (i.e. high MVPA and low screen time). Non-compliance with either or both the MVPA and screen time recommendations therefore explains why so few participants met all of the 24-h Canadian movement guideline. The identification of these groups accords with a recent systematic review [38]. Parker et al., 2019, showed that there are two commonly reported clusters in youth which represent (1) high physical activity and low sedentary time (i.e. *compliers*) and (2) low physical activity and high sedentary time (i.e. *non-compliers*). In our study, at least half of all youth fit into one of these two trajectories. In addition, evidence suggests that these behaviours are interrelated [39]. Studies that have investigated the effect of replacing sedentary time with MVPA have reported positive health implications for youth, including decreased adiposity [40, 41], improved cardiometabolic health [42], and greater fitness [43, 44]. For example, reallocating 10 min of sedentary time to MVPA was associated with a 2.2% reduction in triglycerides in teens [42]. Similarly, reallocating 15 min of sedentary time to MVPA was associated with a 1.3 ml·kg·min^− 1^ increase in VO_2_ peak in children [44]. In addition, displacing an equal amount of sedentary time for MVPA was associated with further long jump and greater flexibility in youth [43].

A group of youth described as *decliners* was also similar among both girls and boys. These youth, which represented around 20% of the sample, displayed a general decline in the probability of attaining both MVPA and screen time recommendations. This aligns with literature indicating general trends of decreasing MVPA [6, 8] and increasing screen time during adolescence [9, 10]. Given that health-related behaviours often take shape during adolescence and track into adulthood [45, 46], efforts should be made to target youth who are at-risk of high screen time and low MVPA. In addition to *non-compliers* described above, youth categorized as *decliners* would also be likely to benefit from interventions replacing sedentary time with MVPA. Since these two groups (*decliners* and *non-compliers*) account for approximately 60% of youth in our sample, intervention strategies targeted to these high-risk individuals would have the potential to reach a large segment of this population. Interventions targeted to these high-risk groups could be facilitated by identification of predictors of group membership such that future studies should investigate predictors of the co-development trajectories identified herein.

Although girls and boys had three similar trajectory subgroups, there was a fourth in each sex that differed. Among girls, a group was identified as being compliers with the screen time recommendation, but not with the MVPA recommendation. Conversely, the fourth group of boys included youth with a high probability of meeting the MVPA recommendation, but also displayed more screen time than recommended. This difference between girls and boys may demonstrate a sex-specific preference for a particular behaviour. It is well established that boys are more active than girls over childhood and adolescence [47, 48]. In addition, boys spend more time on screens than girls [49–51]. These findings are confirmed by ours and two Canadian studies assessing guideline adherence among youth that found that boys are more active and take part in more screen time than girls [36, 37]. Therefore, the *screen-complier* group among girls and the *MVPA-complier* group among boys might represent natural development between the sexes that warrants further study. In addition, these findings highlight the continued importance of developing sex-specific interventions [52].

One of the strengths of the current study is the large number of data collection cycles over the 8 year study duration. Such detailed information on MVPA, screen time, and sleep has allowed for the examinations of the co-development of three health-related behaviours. As such, this is the first longitudinal study describing the adherence to the Canadian 24 h-movement guidelines. Considering that the studies describing adherence to the recommendations found in the 24 h-movement guidelines are mainly cross-sectional studies [37, 40, 53], this study advances our understanding of the development of behaviours during adolescence. Although dichotomising continuous measures is associated with loss of information and drawbacks, such as misclassification of individuals close to, but on opposite sides of the recommendation [54], the choice to dichotomise each behavioural measure was made to present a measure aligned with the guidelines’ definitions. Trajectory analysis methods are useful for summarizing complex longitudinal data. Although results presented herein provide insight into how the behaviours of different individuals co-develop, it must be reiterated that the trajectory groups identified represent latent classes which may be specific to our data. In addition, our measures of physical activity, sleep time and screen time are certainly not perfect. Therefore, the self-reported measures used in this study are subject to misclassification of meeting the guidelines or not and social desirability bias, which may lead to an overestimation of physical activity and sleep time and underestimation of screen time [55]. While the use of direct measures might yield results more aligned with actual physical activity, screen time and sleep levels it would not be feasible to accomplish in such a large sample with frequent data collection. Nonetheless, given the proportion of youth attaining guidelines in our study is similar to proportions reported in studies using representative samples of Canadian youth with both self-report measures similar to ours [36] and Actical accelerometers [37], the results presented herein provide additional support for general trends of MVPA, screen time and sleep among Canadian youth. Another limitation inherent to prospective studies is the likelihood that missing data due to losses to follow up influenced the results. This was nevertheless partly mitigated by the analytical approach that includes all study participants, albeit attributing more weight to those who participated in more survey cycles.

Our results suggest that many young people do not follow the commonly assumed course of decreasing MVPA, increasing screen time and decreasing sleep with age. Although it is very uncommon for youth to adhere to the Canadian 24 h movement guideline recommendations over time, some youth displayed a high likelihood of attaining one or more of the behavioural recommendations. Further, patterns of adherence to the recommendations can differ markedly across different sub-groups of youth. Future research should aim to identify the conditions that favour the adoption of 24-h movement guidelines throughout childhood and adolescence. From an intervention perspective, the results suggest that targeted, as opposed to one-size-fits-all, approaches should be employed for health behaviour promotion among youth.

## Data Availability

The datasets generated during and/or analysed during the current study are not publicly available to insure confidentiality and that any secondary analyses correspond to the objectives of the research project but are available from the corresponding author on reasonable request.

## Appendix

**Table 4:** Model fit characteristics for multi-trajectory model analysis in girls of the MATCH study

Trajectory group	n	Estimated (%, 95 CI)	Assigned (%)	APP (%)	OCC	BIC (*n* = 15,694)	BIC (*n* = 491)
2-group model						− 7491.93	− 7459.01
1	148	29.4 (25.4–33.4)	29.0	93.6	14.6		
2	362	70.6 (66.6–74.6)	71.0	97.0	32.3		
3-group model						− 7225.88	− 7175.65
1	314	60.1 (55.8–64.4)	61.6	94.6	35.6		
2	114	22.4 (18.8–26.0)	22.5	91.7	22.4		
3	82	16.1 (12.9–19.3)	16.0	92.9	26.6		
4-group model						− 7131.09	− 7063.53
1	66	14.6 (11.5–17.7)	12.9	87.7	21.4		
2	293	54.8 (50.5–59.1)	57.5	91.5	32.3		
3	108	21.9 (18.3–25.5)	21.2	89.7	26.1		
4	43	8.7 (6.3–11.1)	8.4	93.0	39.3		
5-group model						− 7083.56	− 6998.68
1	203	37.2 (33.0–41.4)	39.8	86.4	25.4		
2	148	30.2 (26.2–34.2)	29.0	85.0	22.7		
3	47	9.5 (7.0–12.0)	9.2	89.5	34.1		
4	68	14.3 (11.3–17.3)	13.3	86.5	25.6		
5	44	8.7 (6.3–11.1)	8.6	91.5	43.1		
6-group model						− 7081.03	− 6978.82
1	209	36.1 (31.9–40.3)	41.0	82.4	23.4		
2	44	10.7 (8.0–13.4)	8.6	73.2	13.6		
3	110	22.4 (18.8–26.0)	21.6	82.2	23.0		
4	71	15.1 (12.0–18.2)	13.9	87.1	33.7		
5	32	7.0 (4.8–9.2)	6.3	90.9	49.8		
6	44	8.7 (6.3–11.1)	8.6	91.5	53.7		
Final model^a^						− 7154.16	− 7105.65
Non-compliers	228	42.5 (38.2–46.8)	44.7	88.8	23.8		
Decliners	85	18.5 (15.1–21.9)	16.7	86.3	18.9		
Compliers	44	9.0 (6.5–11.5)	8.6	94.0	47.0		
Screen-Compliers	153	30.0 (26.0–34.0)	30.0	89.0	24.3		

*Abbreviations*: *APP* Average posterior probability of classification, *OCC* Odds of correct classification, *BIC* Bayesian information criterion

^a^Variations between the distribution of participants in trajectory group results and final model results to facilitate trajectory group presentation

**Table 5:** Model fit characteristics for multi-trajectory model analysis for boys of the MATCH study

Trajectory group	n	Estimated (%)	Assigned (%)	APP (%)	OCC	BIC (*n* = 15,694)	BIC (*n* = 491)
2-group model						− 5791.23	− 5758.87
1	268	63.5 (58.9–68.1)	64.9	95.0	19.0		
2	145	36.6 (32.0–41.2)	35.1	95.4	20.7		
3-group model						− 5637.29	− 5587.89
1	244	57.0 (52.2–61.8)	59.1	94.4	34.2		
2	60	14.7 (11.3–18.1)	26.8	86.5	13.0		
3	109	27.4 (23.1–31.7)	13.4	95.0	38.6		
4-group model						− 5608.61	− 5542.18
1	68	17.7 (14.0–21.4)	16.5	79.5	11.6		
2	206	47.5 (42.7–52.3)	49.9	89.2	24.8		
3	34	9.4 (6.6–12.2)	8.2	90.0	27.0		
4	105	25.4 (21.2–29.6)	25.4	92.3	36.0		
5-group model						− 5572.02	− 5488.56
1	55	14.8 (14.0–21.4)	13.3	78.2	14.3		
2	179	40.9 (36.2–42.0)	46.2	85.4	23.4		
3	60	14.7 (11.3–18.1)	14.4	86.0	24.6		
4	86	21.7 (17.7–25.7)	21.3	90.1	36.4		
5	33	7.9 (5.3–10.5)	7.2	86.2	25.0		
6-group model						− 5569.36	− 5468.86
1	53	13.4 (10.1–16.7)	12.8	75.1	15.0		
2	166	37.3 (32.6–42.0)	40.2	82.8	24.0		
3	55	15.2 (11.7–18.7)	13.3	80.8	21.0		
4	85	20.9 (17.0–24.8)	20.6	89.1	40.8		
5	25	6.2 (3.9–8.5)	6.0	88.1	36.9		
6	29	6.9 (4.5–9.3)	7.0	85.7	29.9		
Final model*						−5572.59	− 5524.89
Non-compliers	189	42.3 (37.5–47.1)	45.8	86.9	19.9		
Decliners	86	22.6 (18.6–26.6)	20.8	83.8	15.5		
Compliers	47	12.1 (9.0–15.2)	11.4	87.9	21.8		
MVPA-compliers	91	23.0 (18.9–27.1)	22.0	91.7	33.1		

*Abbreviations*: *APP* Average posterior probability of classification, *OCC* Odds of correct classification, *BIC* Bayesian information criterion

**Variations between the distribution of participants in trajectory group results and final model results to facilitate trajectory group presentation
